# Editorial: Frontiers in Phytolith Research

**DOI:** 10.3389/fpls.2020.00454

**Published:** 2020-04-17

**Authors:** Martin J. Hodson, Zhaoliang Song, Terry B. Ball, Rivka Elbaum, Eric Struyf

**Affiliations:** ^1^Department of Biological and Medical Sciences, Faculty of Health and Life Sciences, Oxford Brookes University, Oxford, United Kingdom; ^2^Institute of the Surface-Earth System Science, Tianjin University, Tianjin, China; ^3^Department of Ancient Scripture, Brigham Young University, Provo, UT, United States; ^4^R.H. Smith Institute of Plant Sciences and Genetics in Agriculture, The Hebrew University of Jerusalem, Rehovot, Israel; ^5^Department of Biology, Global Change Ecology Centre, University of Antwerp, Wilrijk, Belgium

**Keywords:** phytolith, silica, silicon, biomineralisation, biogeochemistry, carbon sequestration

Interest in phytoliths has grown significantly in recent years. The Research Topic is unusual in its highly interdisciplinary nature, and in the huge range of scales covered: from cellular and molecular studies of phytolith formation to investigations focussing on the role of phytoliths in biogeochemical cycling. This *Frontiers in Phytolith Research* Topic includes high quality work across this whole range of phytolith research.

For phytoliths to form, plants need to absorb silicon (Si) from their environment. Since Ma et al. (2006) first described a Si transporter in the rice root, considerable interest was raised in establishing the molecular basis of plant Si uptake. The Bokor et al. paper in our issue reports a Si transporter in date palm for the first time. Two papers in our collection (Sun et al.; Li et al.) investigate the effects of Si fertilization on phytolith accumulation in rice, and in both cases showed significant increases in deposition. Whether Si transporters are directly involved in the formation of phytoliths remains to be studied.

Plant internal processes and structure also impact on phytoliths. Phytoliths not only vary in shape and size, but also in their chemistry, and this is influenced by the environment in which they form (Hodson, 2016). Carole Perry has worked on the chemistry of silica deposition in plants for many years, and we were pleased to include a paper from her group (Volkov et al.) in our collection. The authors investigate silica and its carbohydrate matrix in the elaters of *Equisetum arvense*, using Raman and scanning electron microscopy, assisted by density functional theory. Phytolith chemistry has usually been analyzed in bulk samples, but Zancajo et al. investigate individual phytoliths in the leaves of *Sorghum bicolor* using Raman and synchrotron FTIR microspectroscopies. They show that bilobate silica cells have a different silica molecular structure and type of occluded organic matter compared with prickles and long cells.

One of the areas of phytolith research where we have seen major advances in the last 20 years is morphometrics. This work was further advanced by the publication of the International Code for Phytolith Nomenclature (ICPN) 2.0, while we were in the midst of compiling our collection [International Committee for Phytolith Taxonomy (ICPT), 2019]. This will allow phytolith researchers to accurately describe the morphotypes they find in their work, and to compare their results with scientists around the world. Not surprisingly, anatomical and morphometric research feature strongly in five of the papers we received. Both Ge et al. and Bhat et al. worked on members of the Panicoideae. Ge et al. consider morphological variation in the phytoliths from the inflorescence bracts of 38 weed and crop species in China, while Bhat et al. work on the leaf and synflorescence phytoliths of three *Setaria* species. In both cases the authors report that it is possible to distinguish fairly closely related taxa using phytolith morphological and morphometric traits. Two papers take very different approaches to the study of palm phytoliths (Bokor et al.; Huisman et al.). Bokor et al. work on the phytoliths found in stegmata cells present in roots, stems and leaves. The stegmata are located on the outer surface of sclerenchyma bundles or associated with the vascular bundles. Huisman et al. study the phytoliths of 12 palm species from mid-elevation Andean forests and identify a number of distinctive morphotypes that are characteristic of a particular species. But phytoliths do not always distinguish between related taxa. Wang et al. show that bulliform phytolith size could not be reliably used to distinguish between cultivated rice and three wild rice species, and moreover hydrothermal factors (higher temperature, precipitation and water level) led to increased size.

Plants deposit phytoliths for many reasons. It has been known for many years that plant silica acts as a physical defense against grazing and pathogens. Mir et al. added to this body of literature, showing that Si fertilization of rescuegrass decreases herbivory by a grasshopper. They went on to show that the increased silica content of the plants caused greater mandibular wear of the grasshoppers.

When plant organs die and drop to the ground, they then rot and release phytoliths into the soil. Once released two key processes are important: migration of the phytoliths within the soil profile; and breakdown and dissolution of phytoliths. Liu et al. study the translocation of phytoliths in soil profiles in Northeast China. They find that 22% of phytoliths are translocated beneath the surface, and that translocation depends on phytolith size and aspect ratio. The authors suggest that phytolith translocation should be considered in investigations concerning palaeoclimate and palaeovegetation reconstructions. Strömberg et al. (2018) assessed translocation processes within the soil, but then went on to consider the dissolution and breakdown of phytoliths. The major factor in increasing phytolith solubility was geometric surface to bulk ratio. One area they did not cover was the chemical makeup of the phytoliths, and particularly any differences in the breakdown of cell wall and lumen phytoliths. Hodson reviews this topic and concludes that there is no evidence in the literature that cell wall phytoliths were either more or less soluble.

Phytoliths have found applications in many aspects of ecological work, and Solomonova et al. included in our collection is one example. These authors consider the influence of moisture and temperature on the phytolith assemblages of ecosystems in the Altay Mountains. They are able to distinguish between seven of 13 regionally important plant communities by using aggregated and more detailed phytolith morphotypes. For six communities there is too much overlap in their phytolith morphotypes. This kind of work on modern systems is needed before attempting to reconstruct past ecosystems using phytolith assemblages. Although many papers in our collection will be of use to those working in palaeoecology, palaeoclimatology, and archaeology, unfortunately the major gap in *Frontiers in Phytolith Research* are studies looking at using phytoliths to reconstruct past ecology, climates or human activities. Readers are referred to Ball et al. (2016) and Strömberg et al. (2018) for recent reviews of work in these areas.

It was Conley (2002) who first emphasized the importance of phytoliths as a sizable pool of Si in the terrestrial biogeochemical cycle. Because biogenic silica is more soluble than other mineral components of the soil (e.g., aluminosilicates) in most subsequent investigations it has been shown to be a significant source of Si for plant uptake. Our collection includes two papers concerning the role of phytoliths in Si cycling. Gewirtzman et al. investigate the effects of soil warming on cycling of Si in a temperate forest. They find that warming increases Si uptake by vegetation and accelerates the internal cycling of silica. In contrast Koné et al. find that biogenic silica storage in the sediments of Ivory Coast lagoons is dominated by diatom frustules and sponge spicules rather than the phytoliths produced by the abundant macrophytes. They conclude that the macrophytes contribute little to biogenic Si storage in sediments but speculate that fragile phytogenic silica structures may affect local silica cycling.

Parr and Sullivan (2005) first suggested that the carbon occluded within phytoliths (so-called PhytOC) might be significant in the global carbon cycle, and that sequestration within phytoliths might have some potential for tackling climate change. Their work created a whole new sub-discipline in phytolith research and it was not surprising that five of the papers submitted to *Frontiers in Phytolith Research* touched on this area. Two papers (Li et al.; Sun et al.) investigate the effects of fertilization on carbon sequestration in phytoliths from rice. In both cases fertilization has no effect on the carbon content of phytoliths, but it did increase the mass of phytoliths in the plants, and hence the total amounts of carbon sequestered. A further two papers (Chen et al.; Zhang et al.) emphasize the importance of bamboo in carbon sequestration. Chen et al. work on the belowground biomass of monopodial bamboo species in China, and find that this represents an important and overlooked PhytOC stock. Zhang et al. carry out a wider scale investigation of carbon sequestration in phytoliths in the forests of China. They find that sequestration is particularly high in bamboo, and that the litter layer beneath bamboo plants is very high in PhytOC. This could make a very significant contribution to the long term global biogeochemical carbon sink.

In recent years the whole topic of carbon sequestration in phytoliths has become mired in controversy. Some (e.g., Song et al., 2016) are convinced that the original hypothesis of Parr and Sullivan (2005) is correct, and that PhytOC is a highly important store of carbon on a global scale. Others (e.g., Reyerson et al., 2016) consider that carbon sequestration is not significant. The key issue is the extraction procedure used to prepare phytoliths for analysis. Strong extraction may remove carbon from within phytoliths giving low values for PhytOC, and then apparently poor sequestration on a global scale. Weak extraction may leave contaminants on the surface of phytoliths and lead to overestimation of sequestration. Hodson assesses this whole controversy, and attempts to find a way forward. He suggests that cell wall phytoliths are much richer in PhytOC than lumen phytoliths, as demonstrated by Zancajo et al., and that they may be highly significant in global carbon sequestration. Two hypotheses are advanced, one to explain what happens to phytoliths when they are prepared in the laboratory for analysis, and the other what happens in the soil. Hodson concludes that phytoliths probably are an important global carbon store.

The carbon dating of phytoliths has become another controversial area in phytolith research. Discrepancies in dating have been suggested to indicate that it is not a reliable technique, and some workers have suggested the “old carbon hypothesis” to explain these problems (Reyerson et al., 2016). Essentially this involves carbon being taken up from the soil and then selectively deposited in phytoliths. As this carbon will have an older date than that coming from the atmosphere it is postulated to cause problems with dating. However, others (e.g., Piperno, 2016) are critical of this idea and believe the dating problems are due to methodological issues. Zuo and Lu provide a comprehensive review of this topic. They are critical of the “old carbon hypothesis” and suggest that dating of phytoliths often gives consistent results.

Phytolith research is multidisciplinary and undertaken at many different scales. Often work in one area of research throws light on a topic at a different scale. So it is quite possible that the work of Zancajo et al. which suggests that bilobate silica cells in sorghum leaves have a different type of occluded organic matter compared with prickles and long cells may yet prove important when we consider carbon sequestration and dating. Therefore, phytolith researchers need to be aware of work that is some way from their immediate field of research. If this does not happen then we will all miss out. In his opinion article, Katz suggests that we need to break down the disciplinary barriers within phytolith research to produce a superdiscipline. He ends by stating, “Hence, embedding superdisciplinary thinking in plant silicon and phytolith research can not only advance our field, but increase its impact in the merger of Earth and life sciences into a single superdiscipline. Working toward this goal is a true new frontier for plant silicon and phytolith research, for Earth-life sciences and for science in general.” There is much to be said in favor of this idea. We hope that *Frontiers in Phytolith Research* has, in some way, contributed to advancing the superdiscipline.

## Author Contributions

MH wrote the first draft of the editorial. ZS, TB, RE, and ES all commented on the draft. All authors agreed with the final draft.

## Conflict of Interest

The authors declare that the research was conducted in the absence of any commercial or financial relationships that could be construed as a potential conflict of interest.

## References

[B1] Ball T. Chandler-Ezell K. Dickau R. Duncan N. Hart T. C. Iriarte J. (2016). Phytoliths as a tool for investigations of agricultural origins and dispersals around the world. J. Archaeol. Sci. 68, 32–45. 10.1016/j.jas.2015.08.010

[B2] Conley D. J. (2002). Terrestrial ecosystems and the global biogeochemical silica cycle. Glob. Biogeochem. Cycles 16:1121 10.1029/2002GB001894

[B3] Hodson M. J. (2016). The development of phytoliths in plants and its influence on their chemistry and isotopic composition. Implications for palaeoecology and archaeology. J. Archaeol. Sci. 68, 62–69. 10.1016/j.jas.2015.09.002

[B4] International Committee for Phytolith Taxonomy (ICPT) (2019) International Code for Phytolith Nomenclature (ICPN) 2.0. Ann. Bot. 124 189–199. 10.1093/aob/mcz064. 31334810 PMC6758648

[B5] Ma J. F. Tamai K. Yamaji N. Mitani N. Konishi S. Katsuhara M.. (2006). A silicon transporter in rice. Nature 440, 688–691. 10.1038/nature04590 16572174

[B6] Parr J. F. Sullivan L. A. (2005). Soil carbon sequestration in phytoliths. Soil Biol. Biochem. 37, 117–124. 10.1016/j.soilbio.2004.06.013

[B7] Piperno D. R. (2016). Phytolith radiocarbon dating in archaeological and paleoecological research: a case study of phytoliths from modern neotropical plants and a review of the previous dating evidence. J. Archaeol. Sci. 68, 54–61. 10.1016/j.jas.2015.06.002

[B8] Reyerson P. E. Alexandre A. Harutyunyan A. Corbineau R. Martinez De La Torre H. A. Badeck F. (2016). Unambiguous evidence of old soil carbon in grass biosilica particles. Biogeosciences 13, 1269–1286. 10.5194/bg-13-1269-2016

[B9] Song Z. McGrouther K. Wang H. (2016). Occurrence, turnover and carbon sequestration potential of phytoliths in terrestrial ecosystems. Earth Sci. Rev. 158, 19–30. 10.1016/j.earscirev.2016.04.007

[B10] Strömberg C. Dunn R. Harris E. Crifò C. (2018). Phytoliths in paleoecology, in Methods in Paleoecology: Reconstructing Cenozoic Terrestrial Environments and Ecological Communities, eds Croft D. A. Su D. F. Simpson S. W. (Berlin: Springer), 235–287. 10.1007/978-3-319-94265-0_12

